# Pulsatilla

**Published:** 1898-12

**Authors:** 


### HOMŒOPATHIC PHYSICIAN,

A MONTHLY JOURNAL OF

HOMŒOPATHIC MATERIA MEDICA AND CLINICAL MEDICINE.

"If our school ever gives up the strict inductive method of Hahnemann, we are lost, and deserve only to be mentioned as a caricature in the history of medicine."—CONSTANTINE HERING.

Vol. XVIII. DECEMBER, 1898. No. 12.

### EDITORIAL.

PULSATILLA sufferings are made worse from letting the affected limb hang down. This is the grand characteristic of Puls., and it is Dr. Guernsey's keynote. Conium is the reverse, the patient is better from letting the affected limb hang down.

Here are some other notes appropriate to this symptom of letting the affected limb hang down. Some are from Dr. Lippe and some independently collected:

Indium-metallicum, pulsation in the foot from letting the limb hang down.

Natrum-carb., aggravation from letting the diseased limb hang down.

Calcarea-carb., epilepsy aggravated by letting the limbs swing or hang down.

Conium, periostitis, with throbbing, burning pain, ameliorated from letting the diseased limb hang down.

Calcarea-carb., ulcers better when keeping the diseased limb elevated.

Pulsatilla has burning, stinging pains. This recalls Apis and Rhus-tox.

Pulsatilla has bruised pains apparently in the bones.

Pulsatilla has rheumatic pains shifting rapidly from one place to another, or from one side to the other.

Lac-caninum has pains which leave one side and reappear in the same place on the other side; then vanishing from the latter place to again appear in the former position.

Lachesis, the pains go from left side to right.

Lycopodium, from right side to left. These last two indications have been repeatedly given before.

Pulsatilla has chlorosis in company with menstrual symptoms.

Pulsatilla is indicated in intermittent fever when coldness predominates. When thirst occurs before the paroxysm comes on. It seldom happens during the fever.

Pulsatilla has rhagades like Sulphur.

The Pulsatilla pus is yellowish green.

Pulsatilla has inflamed varicose veins.

Varicose veins on thighs occurring during pregnancy indicate Ferrum.

Pulsatilla is indicated in slow, phlegmatic, good-natured people. This is its characteristic. It is one of Dr. Guernsey's keynotes.

Pulsatilla is indicated in timid people. This is a characteristic.

Pulsatilla is indicated in people who are easily reduced to tears. This is Dr. Guernsey's keynote.

Pulsatilla is generally indicated in the disorders of pregnancy. This is Dr. Guernsey's keynote.

Pulsatilla generally has no thirst. This is not, however, an invariable rule.

Pulsatilla and Rhus-tox. both have restlessness. The patient lies still for a little while, but soon he must change to a new position. The Rhus-tox. patient is relieved by this change to a new position, for a few minutes. Under Puls. the change does not bring relief.

Puls. and Rhus both have aggravation when first beginning to move, but better after continuing the motion.

The Pulsatilla patient avoids the rays of the sun because of aggravation from heat.

Pulsatilla has amelioration from lying on the painful side; from eating cold food; from vegetable diet; in cold weather, and from uncovering.

It will be remembered that Bryonia has amelioration from lying on the painful side. It is one of the characteristics of Bryonia.

Phosphorus has amelioration from eating cold food. This is a characteristic of Phosphorus.

Pulsatilla has amelioration from walking slowly in the open air. This is a characteristic of Pulsatilla.

Here are some of Dr. Guernsey's keynotes for Pulsatilla:

Suitable for females with blue eyes, affectionate disposition, and easily excited to tears.

Cannot sleep in the early part of the night. Sleeps late in the morning.

No thirst. Symptoms worse toward evening.

Relieved in the open air, and worse on returning to a warm, close room.

Erysipelatous affections change from one part to another, inclining to spread far around on the buttocks and thighs.

Hemorrhage from erectile tumors. The blood changes in its appearance. It is more apt to flow in the daytime when walking. It is intermittent in its flow.

The patient is moved to tears in giving her symptoms. Very bad taste in the mouth early in the morning. Scanty urine. Craves fresh, cool air.

Pressure in the abdomen and in small of back as if from a stone, with disposition of lower limbs to go to sleep when sitting and attended with ineffectual desire to go to stool.

Menstrual colic with great restlessness—tossing in every possible direction.

Nothing tastes good and she is pale and feeble.

Burning leucorrhœa, thin and acrid. Milky leucorrhœa, with swelling of the vulva, particularly after the menses. Leucorrhœa of thick white mucus, especially when lying down, before and during the menses.

Tension and contraction in the abdomen as if the menses would appear.

Nausea and sometimes vomiting of mucus. Semi-lateral headache and nightly diarrhœa. Symptoms very changeable. Easily moved to tears or laughter. Very well one minute, very miserable the next. Timid and fearful, and yet very mild, gentle, and yielding. Sometimes silent and melancholy.

Nothing tastes good, or she has no sense of taste.

Menses suppressed or else flowing intermittently.

Lumps appear in the breasts of young girls. They are sometimes very painful, affecting the arm of the same side.

Difficulty of breathing after slight emotions.

Constant chilliness even in summer when warmly clad.

Vertigo. Throbbing headache; pressure in the stomach; pain in uterus; dysuria; bad effects from wet feet; ophthalmia; nervous debility; morning sickness; pain so violent that she tosses in every direction with cries and tears.

Menstrual blood thick and dark, or pale and watery; flowing by fits and starts. Menorrhagia; metrorrhagia; profuse at times, at other times intermitting and mixed with clots. Most profuse in persons given to reveries, and in those at the critical age.

Pulsations in the pit of the stomach.

Bad taste in the mouth in the morning on waking. Has to wash it out immediately, because it is so bad she cannot bear it. Does not relish water and does not drink her usual quantity. Nightly diarrhœa. Watery diarrhœa, at night. Sometimes it is unconsciously evacuated. Discharge of blood and mucus during stool. Pallid face. Disposition to faint.

